# Immunotropic Effects of Steroid Hormone Medicines in Combination with Plasma-Treated Solution in Women of a Reproductive Age and Postmenopausal Women

**DOI:** 10.3390/medsci13040209

**Published:** 2025-09-24

**Authors:** Tatyana Ivanovna Pavlik, Nadejda Maximovna Kostukova, Darya Andreevna Razvolyaeva, Evgeny Mikhaylovich Konchekov, Leonid Viktorovich Kolik, Namik Guseinaga-ogly Gusein-zade, Nikolai L’vovich Shimanovskii

**Affiliations:** 1 Department of Molecular Pharmacology and Radiobiology named after P. V. Sergeev, Pirogov Russian National Research Medical University, Moscow 117513, Russia; 2 Prokhorov General Physics Institute of the Russian Academy of Sciences, Moscow 119991, Russiaeukmek@gmail.com (E.M.K.);; 3 Institute of Physical Research and Technology, Peoples Friendship University of Russia (RUDN University), Moscow 117198, Russia

**Keywords:** plasma treated solution, progestins, dexamethasone, postmenopause, cytokines

## Abstract

**Background:** Steroidal glucocorticoid and gestagenic drugs and cold plasma-treated solutions (PTSs) are known to exert anti-inflammatory effects by influencing the production of a number of cytokines. The aim of this work was to test their independent and combined effects exerted on the production of cytokines IL-1, IL-6, TNF-α, TGF-β, and IL-10 and reactive oxygen and nitrogen species (RONS) by leukocytes in women of a reproductive age and postmenopausal women. **Methods:** ELISA and chemiluminescence methods were used for this purpose. **Results:** PTS reduced IL-6 and RONS production by 50% and increased IL-10 production 2-fold in postmenopausal women, and it reduced IL-6 production by 80% and RONS production by 50% in women of reproductive age. When PTS and steroid hormonal drugs are used together, there is a general suppression of cytokine and oxidant activity. **Conclusions:** PTS reduces the production of inflammatory factors by leukocytes and stimulates the production of anti-inflammatory factors, more so in postmenopausal women. Progestins showed greater suppression of pro-inflammatory cytokine and RONS formation and stimulation of anti-inflammatory cytokines for women of reproductive age and dexamethasone showed such results for postmenopausal women.

## 1. Introduction

The regulation of inflammatory processes remains a relevant issue in modern medicine. In clinics, steroid hormones are often used as anti-inflammatory drugs, primarily glucocorticoids [1]. The activation of glucocorticoid receptors suppresses T cell proliferation and activation of transcription factors such as JAK and STAT, reduces the production of pro-inflammatory cytokines, and increases the production of anti-inflammatory cytokines [2,3]. Progestins are also used as anti-inflammatory drugs [4]. And, although progestins are prescribed primarily to treat endometriosis or to control the immune response during pregnancy, their anti-inflammatory and immunomodulatory properties have attracted the continued attention of scientists and clinicians [5,6]. Progesterone is able to reduce the generation of pro-inflammatory cytokines such as IL-1β, IL-6, TNF-α, and IL-12, as well as the production of chemokines such as MCP-1/CCL2 [5]. Progestins shift T cell differentiation towards T-reg cells, reducing the number of Th1 cells. The progestin medroxyprogesterone acetate (MPA), which showed a cytostatic effect on tumor cells of the mammary gland, endometrium, and ovaries, acted on the immune response ambiguously: it reduced the concentration of IFN-γ, TNF-α, and IL-4 in the blood and increased the activity of CD4+ T-lymphocytes [7]. Its analogue gestabutanoil blocked the opening of mitochondrial pores in mitochondria and promoted the formation of reactive oxygen and nitrogen species (RONS) in cells [8].

The anti-inflammatory effects of steroid hormone drugs are known to depend on the concentration of their own hormones in the bloodstream [9], which can depend on gender and age. The prevalence of atopic dermatitis and asthma in childhood is higher in males and predominates in females after the beginning of the menstrual cycle [10,11]. Androgens decrease posttraumatic expression of pro-inflammatory cytokines in the synovial membrane of the joint, while estrogens increase it. Hormone therapy at menopause reduces pro-inflammatory IL-1β levels, increases the T-helper cell population [12], and favorably affects the course of allergic diseases [13].

Lately, non-drug methods of combating inflammatory diseases have been gaining popularity. For example, plasma discharges in a gas stream can inhibit inflammation and accelerate wound healing [14,15,16]. The basis of this effect is considered to be reactive oxygen and nitrogen species (RONS), which are generated by plasma discharge and interact with cells of damaged tissues and immune factors [17]. Low-temperature plasma-treated aqueous solutions (PTSs) accumulate RONS [18,19], which can stimulate an anti-inflammatory response and tissue regeneration [20,21].

A large variation in the effect of the same substances on leukocytes from different donors has been shown previously [22]. To find out how endogenic steroid hormone levels affect the anti-inflammatory activity of medicines, we compared the response of leukocytes from donors of different age groups to the action of PTSs and steroid hormonal medicines. To compare anti-inflammatory efficacy, the common glucocorticoid dexamethasone, progesterone, and two progestins, MPA and gestabutanoil, were selected.

## 2. Materials and Methods

### 2.1. Materials

The materials used were Hanks’ solution (PanEco LLC, cat. no. P020p, Moscow, Russia), RPMI nutrient medium (PanEco LLC, cat. no. C350, Moscow, Russia), fetal bovine serum (PanEco LLC, catalog no. FB-1001, Moscow, Russia), penicillin–streptomycin (PanEco LLC, catalog no. A073p’, Moscow, Russia), medroxyprogesterone acetate (Chemmed LLC, cat. no. MPG00-100, Toronto, ON, Canada), gestabutanoil (17α-acetoxy-3β-butanoyloxy-6-methylpregna-4,6-dien-20-one, of purity higher than ≥99.0% (HPLC), was provided by the Research Laboratory of Molecular Pharmacology, Moscow), progesterone (HPC Standards, 57-83-0, Cunnersdorf, Germany), dexamethasone (RUE Belpharm, Minsk, Belarus), MTT (thiazolyl blue/tetrazolium bromide, cat. no. CAS-298-93-1(Dia-m), CDH, Delhi, India), dimethyl sulfoxide (DMSO) (Tatkhimpharmpreparaty, cat. no. LSR-003126/08, Kazan, Tatarstan, Russia), ficoll solution (PanEco LLC, cat. no. P053p, Moscow, Russia), luminol (Aldaich Chemistry, cat. no. 521-31-3, Burlington, MA, USA), barium sulfate (Chempack, 7727-43-7, Moscow, Russia), pirogenal (Medgamal, Moscow, Russia), and phytohemagglutinin (PanEco LLC, catalog no. M022, Moscow, Russia).

### 2.2. Receipt of Cold Plasma-Treated Solution (PTS)

Cold plasma treatment of the Hanks’ solution was performed for 5 min using a “CAPKO-JET” source [23,24,25]. The treated solution was added to the wells of a 6-well plate with a diameter of 6 cm in a volume of 5 mL. The treatment was carried out in an atmospheric environment, the distance between the piezoelectric transformer generating the plasma and the surface of the solution was 5 mm, and the average discharge power was 1.5 W. The frequency was 21 ± 0.3 kHz, the final voltage was 5 kV. H_2_O_2_ (0.383 ± 0.02 mM), and NO_2_^−^ (106 ± 8 μM) was registered in the resulting solution [22].

### 2.3. Isolation and Cultivation of Leukocytes

Mononuclear leukocytes from 8 conditionally healthy female donors of reproductive age (20–30 years) and 8 conditionally healthy postmenopause female donors (55–70 years) were used for this work. Donors had no inflammatory or immune diseases and were not on hormone therapy. The privacy rights of human subjects were observed, and informed consent was obtained from the donors for the experiments. The research was approved by Local Ethics Committee of Pirogov Russian National Research Medical University (protocol No 250/2025, 21 April 2025). All procedures were performed in compliance with the Declaration of Helsinki of the World Medical Association.

Blood from the reproductive group was drawn during the follicular phase. Ficoll solution with a density of 1.077 g/cm^3^ was used to obtain mononuclear leukocytes.

### 2.4. Assessment of Leukocyte Viability and Cytokine Activity

The final concentration of hormone medicines (progesterone (PG), medroxyprogesterone acetate (MPA), gestabutanoil (GB), dexamethasone (DS)) in the nutrient medium (when cultured overnight) was 10^−6^ M. The PTS volume was 20% of the nutrient medium volume when cultured in medium and 20% of the final solution volume in the cuvette when chemiluminescence was measured. Cell viability was assessed using the MTT assay based on the reduction in tetrazolium dye by NADPH-dependent and glycolytic enzymes of leukocytes [26]. The concentration of cytokines was assessed after 24 h of cultivation in RPMI nutrient medium supplemented with 10% fetal bovine serum and study medicines. Cytokine generation was further stimulated by the addition of phytohemagglutinin (PHA) at a concentration of 4.5 μM and pyrogenal at a concentration of 2.5 μg/mL. Cytokine concentrations were determined by an enzyme-linked immunosorbent assay (ELISA) using ELISA kits from Cloude-Clone Corp. (SEA124Hu, Houston, TX, USA) and Vector-Best (A-8756, A-8766, A-8768, A-8774, Novosibirsk, Russia).

### 2.5. Assessment of Leukocyte Oxidant Activity

We measured the production of reactive oxygen and nitrogen species (RONS) by mononuclear leukocytes through chemiluminescence [27] and denoted it by the term “oxidant activity”. Before analysis, mononuclear leukocytes isolated from blood on the same day were kept in Hanks’ solution with added active ingredients for 2 h. Then, 400 μL of Hanks’ solution, 200 μL of PTS (or pure Hanks’ solution in the control case), 100 μL of 4.5 mM luminol solution, hormones at a final concentration of 10–6 M, 150 μL of cell suspension, and 30 μL of insoluble barium sulfate suspension used to initiate phagocytic activity were mixed in a cuvette. Chemiluminescence measurements were performed on a Lum1200 chemiluminometer at 37 °C. Oxidant activity was assessed by the maximum chemiluminescence intensity achieved after the addition of barium sulfate to the test sample and by the rate of increase in chemiluminescence intensity.

### 2.6. Statistics

The parameters for each donor were measured 4 times, and then the mean values were compared. Figures show the results as medians for 8 donors and deviations from maximum and minimum values. Statistically significant differences between groups (*p* ≤ 0.05 and *p* ≤ 0.01) were calculated using the Wilcoxon criterion and are shown in the figures with * and **.

## 3. Results

**Figure 1:**
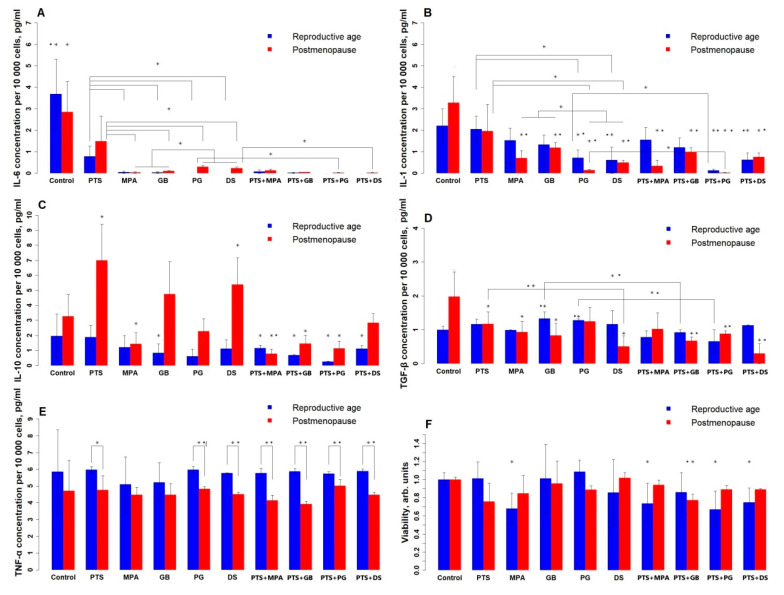
Effects of PTS and steroid hormone medicines on cytokine production and PBMC viability. (**A**)—IL-6; (**B**)—IL-1; (**C**)—IL-10; (**D**)—TGF-β; (**E**)—TNF-α; (**F**)—viability. PTS—plasma-treated solution; MPA—medroxyprogesterone acetate; GB—gestabutanoil; PG—progesterone; DS—dexamethasone. The data are presented as the median in the study sample and the deviations from the maximum and minimum values. The concentrations of progestins and dexamethasone are 1 μM. The symbols * and ** mean a statistically significant difference between samples from the control or a difference between the effects of the agents (*—*p* ≤ 0.025; **—*p* ≤ 0.005). Significant differences between groups (n = 8) were calculated using the Wilcoxon criterion.

Figure 1 shows the change in PBMC viability (1F) and the level of cytokines that they produce (1A–E) under the influence of the tested compounds. Without treatment, there is a difference between the groups only for TGF-β: the reproductive group produces less of it than the postmenopausal group. For TNF-α, no effect of the studied compounds is observed, but its mean level is higher in the reproductive group. PTS reduced IL-6 in both groups (reproductive by 80%, postmenopausal by 50%), reduced TGF-β by 40%, and increased IL-10 2-fold in the postmenopausal group. All studied hormonal medicines reduced IL-6; for the postmenopausal group, PG and DS reduced IL-6 to a lesser extent (90%) than MPA and GB (97–100%). In contrast, IL-1 more intensely reduced PG and DS (PG, DS—90–95%, MPA, GB—70–80%); for the reproductive group, MPA and GB had no significant effect. GB and PG increased TGF-β for the reproductive group by 30%; MPA and GB decreased TGF-β for the postmenopausal group by 50%, and DS decreased TGF-β by 75%. MPA reduces IL-10 in the postmenopausal group by 50%. DS increased IL-10 as much as PTS and reduced TGF-β 2-fold more than PTS in the postmenopausal group. In both groups, PG and DS reduced IL-1 more strongly than PTS (3-fold in the reproductive group and 6-fold in the postmenopausal group), and all hormonal medicines reduced IL-6 more strongly (almost to zero) than PTS. Concomitant administration of PTS and steroid hormonal medicines enhanced the suppression of the generation of all types of cytokines in both groups. The combined use of PTS and DS does not affect IL-10 production in postmenopausal women, whereas separately they increase its production. PTS and GB also separately increase IL-10 production in postmenopausal women, whereas in combination therapy they decrease it. GB and PG increase TGF-β production in the reproductive group, whereas in combination with PTS they decrease it.

This graph takes into account multiple comparison errors, so the error probability was multiplied by 2 and the p-values were reduced to 0.025 and 0.005.

**Figure 2:**
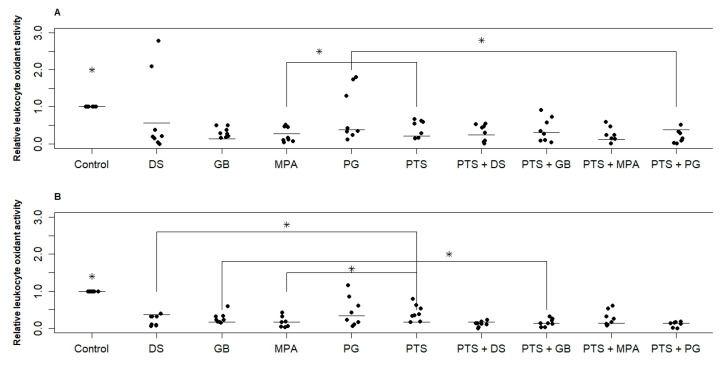
Effect of PTS and steroid hormonal medicines on the oxidative activity (RONS production) of PBMCs. (**A**)—reproductive age; (**B**)—postmenopause. PTS—plasma-treated solution; MPA—medroxyprogesterone acetate; GB—gestabutanoil; PG—progesterone; DS—dexamethasone. Data are presented as median and individual sample data values. The concentrations of progestins and dexamethasone are 1 μM. The symbol * means a statistically significant difference between all samples from the control or a difference between the effects of the agents (*p* ≤ 0.05). Significant differences between groups (n = 8) were calculated using the Wilcoxon criterion.

Figure 2 shows the change in PBMC oxidant activity under the action of the investigated substances for two groups. Both PTS and hormonal medicines significantly reduced RONS production (by 40–60% on average). MPA for both groups and DS for the postmenopausal group reduced RONS production by 20% more than PTS. No significant difference in the effect of different hormonal medicines was found. In the reproductive group, the dispersion of values is significantly greater than in the postmenopausal group. This means that in some women steroid hormones suppress RONS production by white blood cells, and in some women they do not. When PTS and a hormonal medicine (PG and DS for the reproductive group, GB, PG, and DS for the postmenopausal group) are exposed simultaneously, the variance is significantly reduced and suppression of RONS production is more effective.

The variation in parameters within the studied samples is quite large. The error in repeated measurements for each donor was approximately the same and amounted to 20%. This test showed *p* ≤ 0.025 values only when comparing the control with the other samples. When comparing the samples with each other, 0.025 < *p* ≤ 0.05.

## 4. Discussion

**Figure 3:**
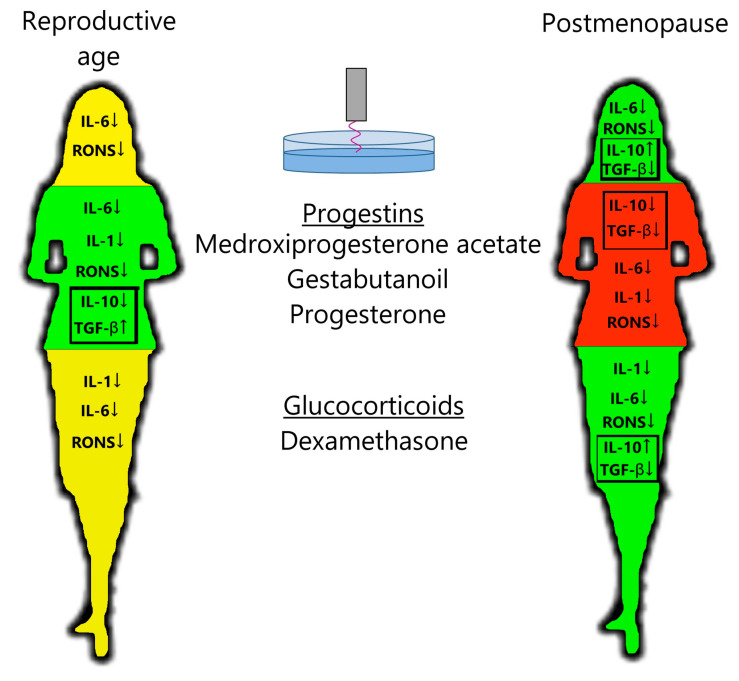
Scheme of action of PTS, progestins, and glucocorticoids on the leukocyte response in women of a reproductive age and postmenopausal women. Green—decrease in inflammatory factors and increase in anti-inflammatory factors; yellow—decrease in inflammatory factors; red—decrease in all immune factors. See description in the text.

PTS decreases leukocyte production of the pro-inflammatory cytokine IL-6 and RONS and increases the production of anti-inflammatory cytokine IL-10 in postmenopausal women (Figure 3), confirming the anti-inflammatory effects of plasma in the clinic. Steroid hormonal medicines suppress the production of pro-inflammatory cytokines and RONS more than PTS, on average. Regarding anti-inflammatory cytokines, in the postmenopausal group, DS stimulates IL-10 production and inhibits TGF-β production. And in the reproductive group, progestins stimulate TGF-β production and have no effect on IL-10 production. When PTS and steroid hormonal medicines are used together, a general suppression of cytokine and oxidant activity is observed. It can also be said that PG affects the cytokine and oxidant response more strongly than its analogs MPA and GB.

When we see inhibition of anti-inflammatory cytokine production, we cannot yet speak of a possible anti-inflammatory effect. When we co-applied PTS and steroid hormones, the generation of all types of cytokines was suppressed, suggesting a general suppressive effect on leukocytes. Therefore, the production of the anti-inflammatory cytokines IL-10 and TGF-β is of great interest.

For the reproductive group, progestins inhibited the production of pro-inflammatory cytokines and RONS and stimulated the production of anti-inflammatory cytokines, whereas dexamethasone only inhibited the production of pro-inflammatory cytokines (Figure 3). In contrast, for the postmenopausal group, dexamethasone had a stronger anti-inflammatory effect than progestins on all parameters studied. Progesterone levels in women of reproductive age are higher, even in the follicular phase, than in postmenopausal women [28]. Blood cortisol levels are independent of age (approximately 10 µg/dL) [29,30,31,32]. The number and affinity of glucocorticoid receptors (GRs) in PBMCs are also independent of age [33,34,35,36,37,38], and the number of membrane progesterone receptors (mPRs) in PBMCs drops to almost zero in postmenopausal women [39]. The number of receptors for cortisol and progesterone in PBMCs at reproductive age appears to be comparable (GRα/GADPH = 0.8–1 [40,41]; mPR/GADPH = 0.5–1 [42,43]). One can assume that thanks to the level of progesterone receptors being comparable to that of glucocorticoid receptors at reproductive age, the action of progestins is predominant. The affinity of these receptors and the activity of their signaling pathways should also be compared. If white blood cells lose their receptors for progesterone in postmenopausal women, then glucocorticoids would become the main active steroids. This hypothesis needs to be tested on a larger sample.

IL-10 production is only stimulated by dexamethasone in the postmenopausal group and not stimulated by progestins in reproductive group. In contrast, TGF-β production was stimulated by progestins in the reproductive group and depressed by dexamethasone in the postmenopausal group. Studies on mice showing that glucocorticoids stimulate leukocyte production of both IL-10 and TGF-β are common in the literature [44,45,46]. However, in a human study by Karagiannidis et al. [47], glucocorticoids were shown to stimulate the production of IL-10 only, but not TGF-β, by T regulatory cells. The results of studies on the effects of progestins on TGF-β production are contradictory: one study [48] reported no effect of a progestin-only contraceptive implant (etonogestrel) on TGF-β levels in the blood of women. In another study [49] on the endometrium, MPA increased TGF-β3 levels during the secretory phase. It is important to continue studying the immunotropic effect of steroid hormonal medicines in different groups of patients.

This work dealt with two small samples, which means there is a risk of statistical error. The standard deviation of leukocyte RONS production for these samples ranged from 0.21 to 0.28 [50,51,52]. If we take the standard deviation of the general population as 0.25, then the power for a sample where n = 8 will be approximately 75%. The results obtained are interesting, and we hope to confirm them in the future on larger samples.

## 5. Conclusions

PTS, like steroid hormone medicines, depresses the production of inflammatory factors by leukocytes and stimulates the production of anti-inflammatory factors, more so in postmenopausal women. It is water-soluble, which can be helpful for its absorption, it has a milder effect than steroid hormones, and it does not have hormone-specific side effects. For women of reproductive age, progestins showed the best anti-inflammatory effect, and for postmenopausal women, dexamethasone showed the best anti-inflammatory effect. This phenomenon is probably due to the different ratio of progestin and glucocorticoid receptors in leukocytes. We hope that this work will be an important step towards personalized medicine.

## Data Availability

The original contributions presented in this study are included in the article. Further inquiries can be directed to the corresponding author.
